# Supplementary material for: Role of Mitochondrial Glycerol-3-Phosphate Dehydrogenase in Metabolic Adaptations of Prostate Cancer
Source: Cells. 2020 Jul 23;9(8):1764. doi: 10.3390/cells9081764 (PMC7464303; doi:10.3390/cells9081764)
Supplement: Supplementary file 1 [file cells-09-01764-s001.zip › Cells_mGPDH Supplementary figures.pdf]

Supplementary figure 1

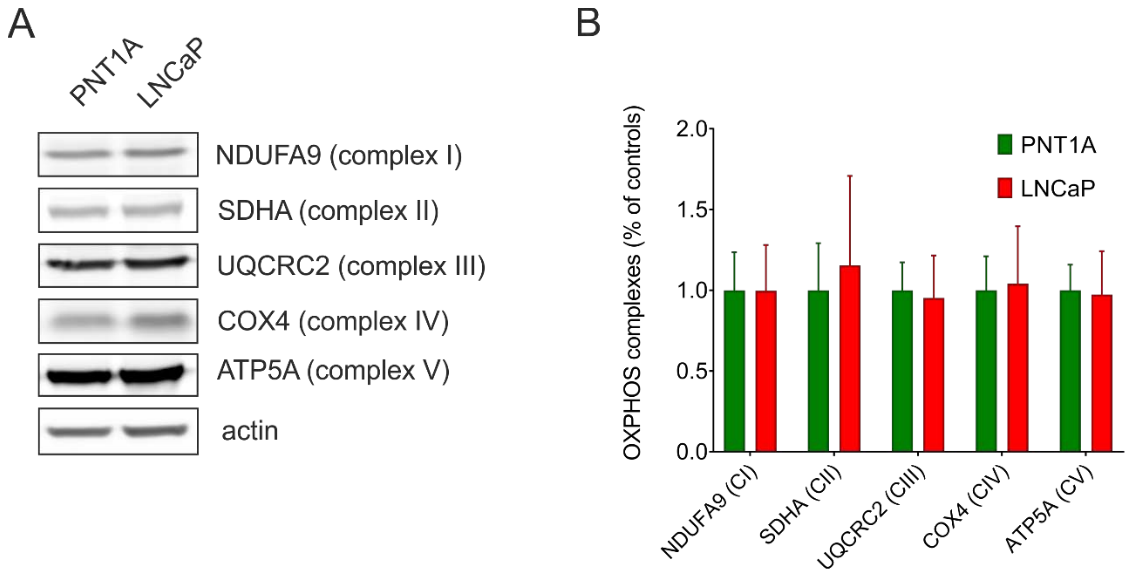

Supplementary figure 1. OXPHOS content in prostate cancer cells. Cell lysates (20 µg protein) were separated on SDS-PAGE and content of representative OXPHOS subunits was analyzed by Western blot using specific antibody against NDUFA9 (complex I), SDHA (complex II), UQCRC2 (complex III), COX4 (complex IV) and ATP5A (complex V). Actin was used as a loading control. (**A**) Representative blot and (**B**) antibody signals quantification normalized to actin levels and expressed as % of control values is depicted. Data represents the means ± S.D. (n=5).

Supplementary figure 2

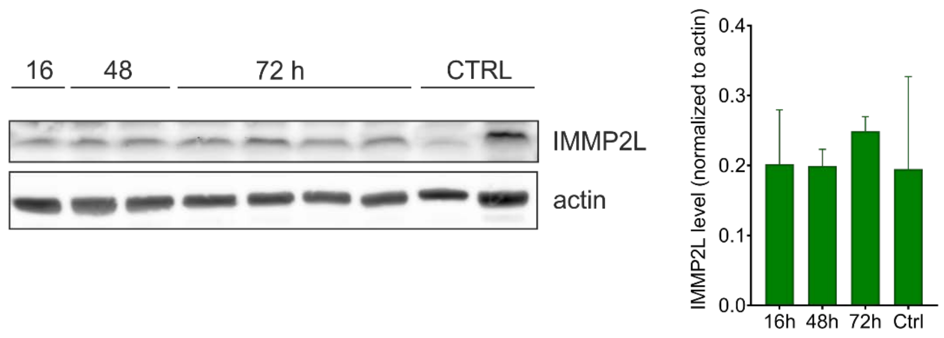

Supplementary figure 2. IMMP2L content. Cell lysates (20 µg protein) of HEK293 cells transiently transfected with vector containing mGPDH with FLAG tag on C-term were separated on SDS-PAGE and content of IMMP2L was analyzed by Western blot using specific antibody. Actin was used as a loading control. Quantified data represents IMMP2L signal normalized to actin and is expressed as means ± S.D. (n=3).

Supplementary figure 3

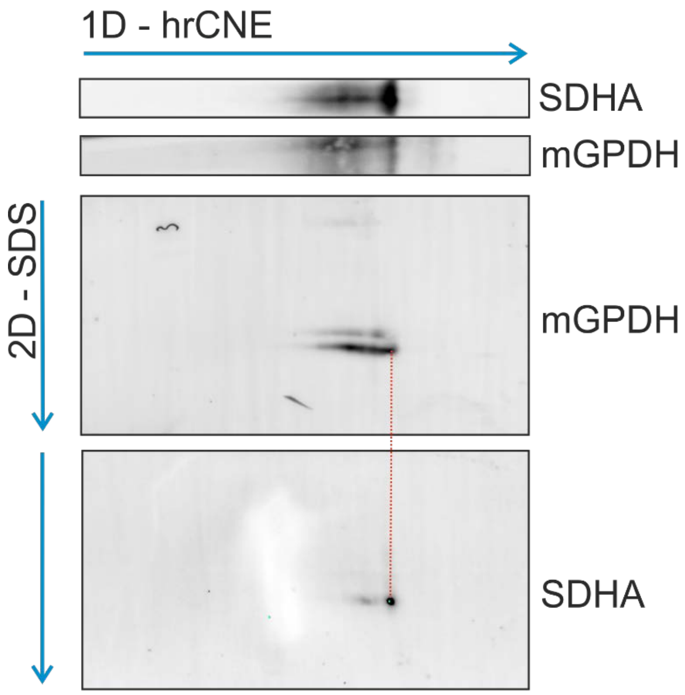

Supplementary figure 3. mGPDH native forms in LNCaP cell line. Cells were solubilized by digitonin (2 g/g) and samples (40 µg protein) were separated on high-resolution clear native electrophoresis (hrCNE, 5-13 %) and either used for Western blot detection of mGPDH and SDHA subunits using specific antibodies, or strips were denatured, separated in second dimension on SDS-PAGE and subsequently analyzed by Western blot as in first dimension.
